# Promethazine Is Increasingly, Inappropriately, Being Prescribed as a Sleep-Aid

**DOI:** 10.1192/bjo.2026.11857

**Published:** 2026-06-30

**Authors:** Jacob King, Kevin Kumar, Kirstie Anderson

**Affiliations:** 1 Central and North West London NHS Foundation Trust, London, United Kingdom; 2 Division of Psychiatry, Imperial College London, London, United Kingdom; 3 NewcastleuponTyne Hospitals NHS Foundation Trust, Newcastle, United Kingdom

## Abstract

**Aims:**

Promethazine is a phenothiazine compound first synthesised in the 1940s, with prominent sedative qualities. As a first-generation antihistamine, promethazine has strong affinity as an antagonist of H_1_histaminergic receptors of the tuberomammillary nucleus, as well as moderate antagonism of muscarinic cholinergic receptors, and weak-moderate affinity to receptors in the serotonergic, dopaminergic, adrenergic and glutaminergic systems. While in the UK promethazine is publicly available ‘over the counter’, it is not recommended by NICE guidelines for insomnia.

**Methods:**

We narratively summarise the available evidence for promethazine’s benefit on human sleep outcomes with structured searches, as well as publicly available prescribing trends data from OpenPrescribing and published academic reports.

**Results:**

Promethazine is increasingly prescribed by primary care physicians and psychiatrists in United Kingdom as a treatment for all kinds of sleep concerns, with a near doubling of English GP prescriptions in the past 5 years. There is no good evidence for the clinical benefit of promethazine on sleep outcomes, with an absence of clinical trials or adequate measures of sleep. Use of promethazine as a sleep-aid is actively advised against in international expert consensuses, with evidence based alternatives available including the NICE guidelines’ first-line option, Cognitive-Behavioural Therapy for Insomnia (CBT-I).

**Conclusion:**

For several reasons, it appears that promethazine is not an appropriate treatment for any flavour of sleep problem. First, because there is no clinical trial evidence. Secondly, it is important to remember that sedation is not sleep. These two states of reduced consciousness are different phenomenologically, physiologically, and appear different on EEG recordings. The multiple benefits of natural human sleep cannot be assumed to be achieved by this kind of artificial sedation. Histaminergic antagonism has a very complex pharmacodynamic profile, and psychiatrists cannot expect that there will be linear dose-effects. Third, the addictive nature of promethazine must be acknowledged: there are significant levels of recreational use of promethazine worldwide, in part because of its effects in potentiating opioid agents. Moreover tolerance develops quickly, with diminished sedative effects seen in as few as 3 days. Withdrawal symptoms are common, with rebound insomnia widely reported after long-term use. Finally, the side effect profile of promethazine is of concern including, QTc prolongation, weight gain, and the potent cholinergic effects have a causal effect on dementia. This poster concludes with a summary of alternative approaches for psychiatrists using promethazine in their practice, including CBT-I and alternative, more sleep problem-specific medication options.

